# *Plasmodium falciparum* Infection Patterns Since Birth and Risk of Severe Malaria: A Nested Case-Control Study in Children on the Coast of Kenya

**DOI:** 10.1371/journal.pone.0056032

**Published:** 2013-02-13

**Authors:** Klara Lundblom, Linda Murungi, Victoria Nyaga, Daniel Olsson, Josea Rono, Faith Osier, Edna Ogada, Scott Montgomery, J. Anthony G. Scott, Kevin Marsh, Anna Färnert

**Affiliations:** 1 Unit of Infectious Diseases, Department of Medicine Solna, Karolinska Institute, Stockholm, Sweden; 2 Kenya Medical Research Institute, Centre for Geographical Medicine Research (Coast), Kilifi, Kenya; 3 Unit of Biostatistics, Department of Epidemiology, Institute for Environmental Medicine, Karolinska Institutet, Stockholm, Sweden; 4 Clinical Epidemiology Unit, Department of Medicine Solna, Karolinska Institutet, Stockholm, Sweden; 5 Clinical Epidemiology and Biostatistics Unit, Örebro University Hospital, Örebro University, Örebro, Sweden; 6 Department of Primary Care and Public Health, Charing Cross Hospital, Imperial College, London, United Kingdom; 7 Centre for Clinical Vaccinology and Tropical Medicine, Churchill Hospital, University of Oxford, Oxford, United Kingdom; Walter & Eliza Hall Institute, Australia

## Abstract

Children in malaria endemic areas acquire immunity to severe malaria faster than to mild malaria. Only a minority of children suffers from severe malaria and it is not known what determines this. The aim of this study was to establish how *P. falciparum* infections during the first years of life affect the risk of severe malaria. A matched case-control study was nested within a large birth cohort set up to study the immunoepidemiology of pneumococci on the Kenyan coast. Infection patterns in three-monthly blood samples in cohort children admitted to hospital with severe malaria were compared to controls matched on age, residential location and time of sampling. *P. falciparum* detected at least once from birth conferred an increased risk of severe malaria and particularly if multiclonal infections, as characterized by genotyping of a polymorphic antigen gene, were ever detected. The results show for the first time that children with severe malaria have more infections early in life compared to community controls. These findings provide important insights on the immunity to severe disease, knowledge essential for the development of a vaccine against severe malaria.

## Introduction

Severe *P. falciparum* malaria continues to cause an intolerably high burden of morbidity and mortality on children living in malaria endemic areas [1]. In areas of high transmission, life-threatening forms of malaria are restricted to young children before they gradually develop clinical protection after repeated exposure. Understanding how immunity to severe manifestations of malaria develops is important in designing interventions to reduce severe disease and death.

The age distribution of severe malaria indicates that resistance to serious complications develops much earlier than immunity to uncomplicated and asymptomatic infections [2]. One possibility is that within an age cohort the individuals who develop severe disease are relatively naïve by virtue of the pattern of exposure to malaria that they experience in early life. The number of infections needed to achieve protection against severe malaria is however not clear. Protection against non-cerebral severe malaria has been proposed to be acquired after only a few infections, and even after a single infection in infants with transferred maternal antibodies [3]. Prospective risk assessments of severe malaria in relation to exposure require large cohorts to capture the relatively rare event of severe disease and have not previously been reported.

Three major syndromes of severe malaria have been identified in children: cerebral malaria severe malarial anemia, and respiratory distress as a sign of acidosis [4]. Cerebral malaria is predominant in areas of low to moderate transmission and peak incidence occurs in children older than 2 years [2], [5], [6]. Cerebral malaria is also one of the most common severe features in previously non-immune adults [7] and in outbreaks of malaria in areas inhabited by a previously un-exposed population [8]. Severe malarial anemia is reported in highly endemic areas affecting mainly young children around the age of 1 year [2]. Respiratory distress with deep breathing has been shown to be a good “proxy” for metabolic acidosis and an indicator of severity [4].

The *P. falciparum* parasite is a highly polymorphic organism with an extensive genetic diversity and natural infections are often composed of several genetically distinct clones expressing different antigen variants. Based on the knowledge from malaria therapy of neuro-syphilis [9] as well as animal models [10], protective immunity to malaria is considered to have a substantial degree of “strain-specificity” reflected by more pronounced clinical symptoms in individuals infected with a new “strain”. Whether immunity to severe malaria develops in a strain-transcending manner remains unclear, as does the number of different parasite variants that needs to be encountered to acquire protection. A well-established method to characterize *P. falciparum* populations is by genotyping the highly polymorphic merozoite surface protein-2 gene (*msp2*), encoding a thoroughly studied potential vaccine candidate antigen [11]. Since the parasite is haploid in the human host, clones i.e. genetically identical sets of blood-stage parasites derived from the same progeny, will have the same *msp2* genotype and different infections can be studied within individuals over time.

The aim of this study was to investigate whether the pattern of exposure and particularly number of infections and different clones in early childhood affect the risk of severe malaria. A large birth cohort established in Kilifi District on the coast of Kenya, offered the opportunity to study the infection patterns from birth in relation to severe malaria outcome. In a nested case-control design we have investigated the parasite prevalence and the number and types of *P. falciparum msp2* genotypes in three-monthly samples collected from birth in cohort children who subsequently were admitted with severe malaria and from controls matched for age, time of sampling and area of residency. Moreover, serum samples were analysed with regards to antibodies to whole *P. falciparum* schizont extract as a marker of previous parasite exposure [12], [13].

## Materials and Methods

### Study Setting

The study was conducted in Kilifi District along the Kenyan coast. Approval for the study was granted by the Kenyan Medical Research Institute National Ethics Committee and the Central Ethical Review Board Stockholm, Sweden. Kilifi District is mesoendemic for *P. falciparum* malaria and transmission typically has two seasonal peaks (June-August and November-December). Spatial differences in transmission intensity and entomological inoculation rate in the area have been described [14] and malaria morbidity and mortality decreased markedly between 2000 and 2007 [15].

Kilifi District Hospital (KDH) serves as a first referral centre for more than 500 000 people. All children admitted to KDH are investigated with a malaria slide and blood cultures, except those admitted after accidents or for elective procedures. Clinical and laboratory data are recorded systematically at admission.

The Kilifi Health and Demographic Surveillance system (KHDSS), established in 2000, covers 891 km^2^ around KDH and currently tracks a population of about 240000 people. All homesteads in KHDSS are visited 4–6 monthly to collect information on births, migrations and deaths [16].

### Study Population and Cohort Visits

A birth cohort, the Kilifi Birth Cohort (KBC) was set up within the KHDSS in 2001 to study the immunoepidemiology of pneumococcal disease. Children either born at KDH or presenting to the vaccination clinic within their first month of life were recruited and subsequently followed with three-monthly visits for two years. The study was explained to parents or guardians by team members fluent in Swahili or Giriama and parents were also given written versions of the information. At each visit, axillary temperature was recorded together with sampling of 2 ml venous blood (without anticoagulant) and a thick and thin film for malaria microscopy. The blood samples were centrifuged and blood clots and serum samples were stored at −80°C. Children unwell during the study visits were attended to by medical staff and referred to KDH. Parents/guardians who did not bring their children for follow-up visits were visited in their homes the following day and again invited to attend the three-monthly sampling.

### Severe Malaria Cases and Controls

A matched case-control study was nested within the Kilifi Birth Cohort. Cases were children admitted to KDH between April 2002 and January 2010 with *P. falciparum* parasites detected by microscopy together with either one or more of the following signs (syndromes) (1) impaired consciousness defined as Blantyre Coma Score <5 [17]; (2) severe anemia defined as hemoglobin <5g/dl [4]; and/or (3) deep breathing and/or chest indrawing i.e. respiratory distress as a sign of acidosis [4], [18]. Children with positive bacterial cultures from blood or cerebrospinal fluid and/or >10 white blood cells in cerebrospinal fluid were excluded to avoid misclassification of children with other infections and incidental parasitemia [19].

Controls were cohort children who had not developed severe malaria up to the time point when the matched case had its episode, and who were individually matched to cases on age (+/−4 months), sampling time (+/−3 months) and residential location. Three controls were sought for each case.

### Detection of *P. falciparum* by Microscopy

Thick and thin blood films were prepared from finger prick blood and stained with Giemsa. Slides were examined under light microscopy and parasites were counted against 200 leukocytes or 500 erythrocytes. *P. falciparum* densities were defined as the number of asexual parasites/µl of whole blood, based on an estimated leukocyte count of 8000/µl or 5 million erythrocytes/µl, respectively. Each blood film was evaluated separately by two expert microscopists and discrepancies resolved by the results from a third microscopist. The slides from the cohort visits were read during 2010. Slides taken at the time of admissions were read promptly as part of the acute clinical management.

### Genotyping of *P. falciparum* Infections

DNA was extracted by processing frozen blood clots by high-speed shaking in a cell disruptor [20] followed by extraction with Puregene kits (Qiagen). Genotyping of the polymorphic block 3 of the *msp2* gene was performed by fluorescent PCR followed by capillary electrophoresis [21]. In brief, the PCR included an initial amplification of the outer *msp2* domain, followed by two separate nested reactions with fluorescent primers targeting the two allelic types of *msp2:* FC27 and IC (also referred to as 3D7), and fragment analysis in a DNA sequencer (3730, Applied Biosystems) and GeneMapper software (Applied Biosystems).

### Antibody Assay

Antibodies against *P. falciparum* schizont extract were assessed by Enzyme-linked immunosorbent assay (ELISA) using the A4 parasite line [12], [13]. Test samples were scored as positive if the OD values were above the mean+3 SD of 20 European sera.

### Statistical Analysis

Statistical analyses were performed using PRISM GraphPad and R version 2.13.1. Parasite prevalence at the cohort visits was based on results from microscopy as well as PCR genotyping of the *P. falciparum msp-2* gene depending on availability of samples. The number of concurrent clones was categorized as 1 clone or ≥2 clones (i.e. multiclonal infections). Patterns of infections in three-monthly samples were defined in three categories; parasite negative in all samples, parasite positive with single clone and parasite positive with multiple clones in at least one sample. Conditional logistic regression, including risk sets where at least one matched control was available for each case, was used to analyze the risk of being a case given previous parasite exposure. This analysis included the 42 risk sets where the case was no older than 2 years and 3 months at the time of severe malaria episode and thus had samples until three months before the admission. The analyses were adjusted for the logarithm of the number of visits, which was approximately linearly related to the logarithm of the odds of becoming a case. An unconditional case-control study was performed within the 61 cases according to syndrome; severe malaria with impaired consciousness versus non-cerebral severe malaria, using an exact logistic regression due to the small sample size, with a model adjusted for age (in six-month categories) and number of visits.

## Results

**Figure 1:**
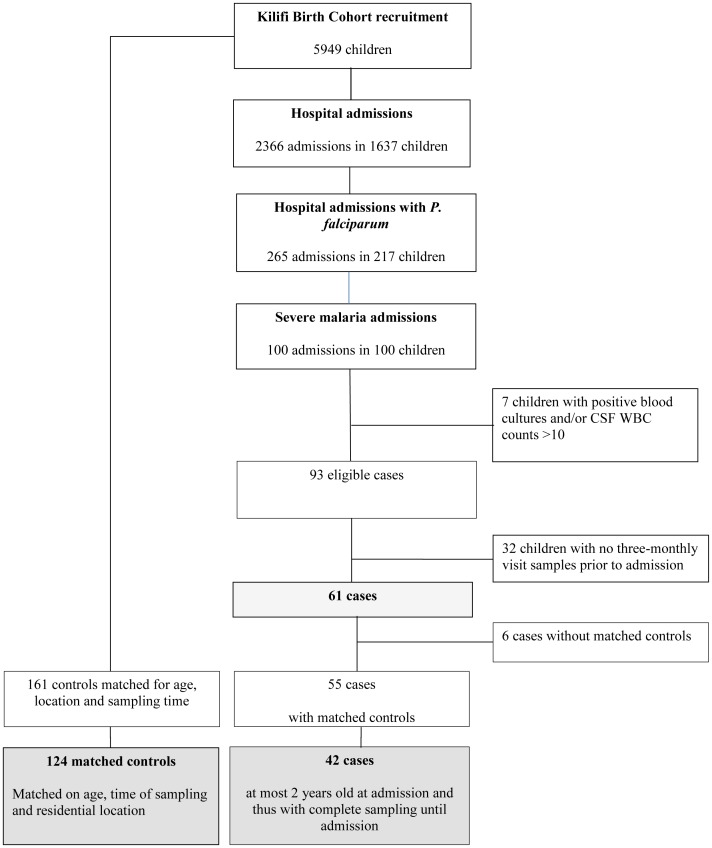
Matched case-control study profile.

**Figure 2:**
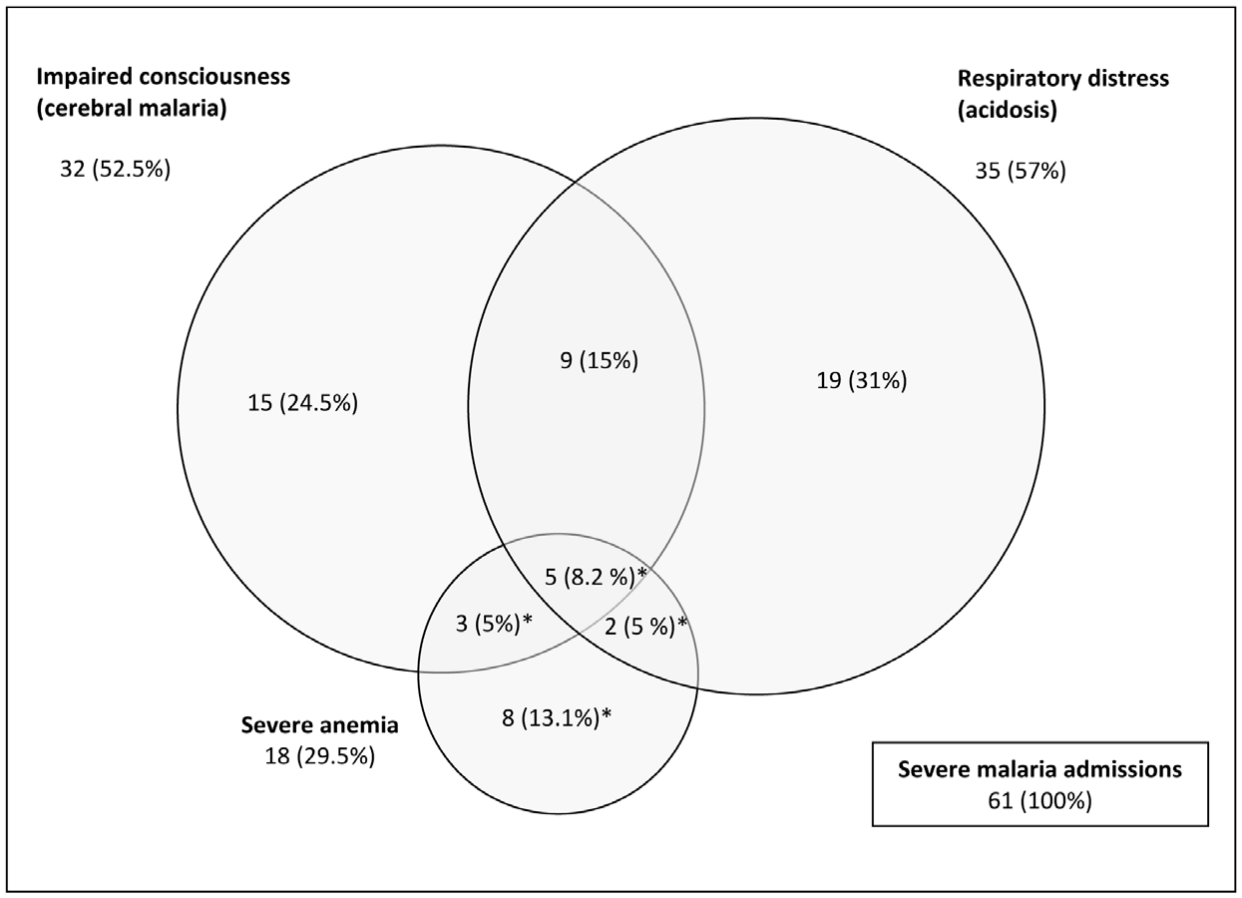
Syndromes overlap and mortality among the 61 cohort children admitted with severe malaria to Kilifi District Hospital. Four children died (marked *).

In total 5949 children were recruited into the Kilifi Birth Cohort between March 2001 and March 2008; of which 1637 children (28%) had been admitted to Kilifi District Hospital until January 2010. In 265 of the total 2366 (11%) admissions, a blood film was positive for *P. falciparum*, and 93 children (4% of all admissions, 35% of admissions with parasites) fulfilled the criteria of severe malaria by a strict syndrome definition (Figure 1). Among these, 61 children had three-monthly samples collected prior to admission; 42 children with one of the three syndromes (impaired consciousness, severe anemia, respiratory distress), 14 with two and 5 children presenting with all three syndromes (Figure 2). No child in the cohort was admitted with severe malaria more than once. Four children died at the time of the severe malaria admission. The median age at admission was 14.8 months (IQR 7.5–24.5) and varied over time (Figure S1) and did not differ between children with or without impaired consciousness. Matched controls were available within the cohort for 55 of the 61 cases (52 cases with 3, 2 cases with 2 and 1 case with 1 control, respectively). The degree of matching is demonstrated in Table 1.

**Table 1:** Characteristics of the study population.

Characteristics	Cases (n = 61)	Controls (n = 161)
Sex female, n (%)	31 (51)	78 (48)
Age (months) at the time of case admission^a^, mean (range) all	19.3 (1.9–37.0)	19.5 (1.6–37.5)
42 risk sets^b^	12.5 (3.9–26.9)	12.5 (2.3–28.2)
Number of three-monthly visits per child, mean (range)	2.77 (1–8)	3.09 (1–8)
Proportion parasite positive visits^c^, n (%)	17/150 (11)	30/432 (7)
Proportion of children with at least one parasite positive visit^c^, n (%)	20/61 (33)	25/161(15)
Proportion of children with at least one antibody positive visit^d^, n (%)	44/61 (75)	105/161 (70)
Visits with fever, n (% of visits)	9/169 (5.3)	27/498 (5.4)
Visits with fever and parasites (by microscopy), n (% of visits)	2/169 (1.2)	10/498 (2.0)

a. age at case admission of the cases and respective controls.

b. restricted to the 42 risk sets where case was younger than 2 years and 3 months at the severe malaria admission and thus had complete follow up periods before admission,

c. including PCR and microscopy results.

d. antibodies to schizont extract detected in at least one of the three most recent visits before admission in the cases and corresponding time in the controls.

### Parasite Patterns at Three-monthly Visits and Severe Malaria Admission

*P. falciparum* parasites were detected by PCR or microscopy in at least one of the three-monthly visits in 33% of the cases and 15% of the controls (Table 1). Among the PCR positive samples, most were composed of a single *msp2* genotype i.e. clone (65% and 70% in cases and controls, respectively). The number of clones in multiclonal infections ranged from 2–3 clones in the cases and 2–6 clones in the controls. The cumulative number of clones, including all samples, ranged from 0–6 in cases and 0–15 clones in controls. The number of clones was not correlated to parasite densities (r = −0.25, *P* = 0.33). Children admitted with impaired consciousness had only one single clone in samples prior to admission; whereas children with respiratory distress and/or anemia (without impaired consciousness) had up to 6 clones cumulatively in three-monthly visits.

Hospital admission samples from the admission sample archives were available for genotyping in 40 of 61 cases. For 21 cases, admission samples had either not been collected or had been used in other studies. Single clones were detected in 52%, and the multiclonal infections were composed of 2 or 3 clones. None of the cases had been infected with the exact same *msp2* allele (same type and fragment size) before admission and 5 of 10 cases had a new *msp2* allelic type (FC27 or IC) at admission. The same alleles (at bp precision) were detected in consecutive three-monthly visits in 3 control children (illustrated in File S1). Additional information of *msp2* genotyping profiles and allele frequencies in visits and admissions are presented in the Supporting information (Figure S2 and File S1).

### Exposure and Risk of Severe Malaria

Detection of *P. falciparum* parasites in at least one of the visits predicted, compared to only negative visits, an increased risk of admission with severe malaria (OR 3.70, 95% CI 1.25–10.92, *P* = 0.018) and particularly if a multiclonal infection was ever detected (OR 15.32, 95% CI 1.49 −157.40 *P = *0.022) (Table 2). Parasite positivity in the most recent sample before admission gave a similar odds ratio as parasite positivity at any time (OR 3.62, 95% CI 0.84–15.71, *P = *0.085). Assessment of the risk of severe malaria with regards to parasite status at different ages, number of parasite positive visits, or to cumulative number of clones was restricted by limited data for subgroup analysis.

**Table 2:** Risk of severe malaria associated with parasite positivity, number of clones and antibodies to schizont extract in three-monthly visits before admission.

Exposure	OR (95% CI)^b^	*P*
Negative in all	1 (ref)	–
Parasite positive in any^c^	3.70 (1.25–10.92)	0.018
Negative in all	1 (ref)	–
At most 1 clone^ d^	4.30 (1.12–16.46)	0.033
≥2 clones in any sample^ d^	15.32 (1.49–157.40)	0.022
Negative in all	1 (ref)	–
Antibody positive in any^ e^	0.76 (0.31–1.89)	0.558

Conditional logistic regression adjusted for log number of visits including 42 risk sets where the case was no older than 2 years and 3 months at the time of severe malaria episode and thus had samples until three months before the admission^a^.

a. excluding 13 risk sets with children with longer time since their last visit (4–47 months).

b. adjusted for log number of visits in the period before the severe admission of the cases and respective periods in the controls.

c. including PCR and microscopy results.

d. including only risk sets where PCR results were available (n = 41).

e. antibodies to schizont extract detected in at least one of the three most recent visits before admission in the cases and corresponding time in the controls, including 40 risk sets where samples for antibody analysis were available.

Antibodies against *P. falciparum* schizont extract (assessed as a marker of ever being exposed) were detected in at least one of the three most recent samples before admission, in 75% of cases and 70% of controls, respectively (Table 1). Children with parasite positive visits were all antibody positive, except five children (one with fever). There was no association between detection of anti-schizont antibodies and severe malaria (OR 0.76, 95% CI 0.30–1.89).

Exposure was further assessed with regards to the different syndromes of severe malaria. Anti-schizont antibodies were detected before admission in three-monthly samples in 59% of children with impaired consciousness and 86% of children with respiratory distress and/or anemia (without impaired consciousness) (Table 3). Within a comparison among the 61 cases with severe malaria the odds of having impaired consciousness was lower if antibodies were ever detected (OR 0.15, 95% CI 0.03–1.23, Table 4).

**Table 3:** Assessment of exposure in three-monthly visits from birth until admission^a^ in 61 children admitted with severe malaria.

		Impaired consciousness^b^ n(%)	Non-cerebral malaria^c^ n (%)
Number of children		32	29
Parasite positive visits	0	25 (78)	19 (65)
	1	7 (22)	8 (28)
	2	0	2 (7)
	Proportion positive	7 (22)	10 (34)
Number of clones in PCR positive visits	Only 1 clone^d^	6 (19)	5 (17)
	Ever multiclonal	0	5 (17)
	Cumulative number^e^, median (range)	1 (1–1)	2 (1–6)
Antibody positive visits	Proportion positive	19/31 (62)	25/28 (89)
Exposed^f^		20/32 (63)	25/29 (86)
Not exposed^g^		12/32 (37)	4/29 (14)

a. samples until 2 years of age at most.

b. cases with impaired consciousness with or without other syndromes.

c. including respiratory distress and severe malaria anemia and not impaired consciousness.

d. ever detected in all samples.

e. total number of clones detected in an individual including all visits.

f. exposed (parasite positive and/or antibodies to schizont extract at least once) n (%).

g. not exposed as determined by PCR and antibodies to schizont extract.

**Table 4:** Risk of impaired consciousness in relation to parasite exposure in three-monthly samples among 61 cases with severe malaria.

Exposure	OR (95% CI)	*P*	
Ever parasite positive^ a^	0.50 (0.08–2.49)		0.46
Ever multiple clones^ b^	0.44 (0–4.91)		0.51
Ever antibody positive^ c^	0.15 (0.03–1.23)		0.06

Unmatched case-control analysis performed with exact logistic regression adjusted for age and number of samples, defining patients with impaired consciousness as “cases” (n = 32) and those with non-cerebral severe malaria as “controls” (n = 29).

a. including PCR and/or microscopy results.

b. including only PCR results.

c. antibodies to schizont extract detected in at least one of the three most recent visits before admission in the cases and corresponding time in the controls.

## Discussion

In this matched case-control study nested within a birth cohort on the coast of Kenya, detection of *P. falciparum* infections, especially infections with multiple clones, in three monthly samples during the two first years of life was associated with an increased risk of admission with severe malaria. The infection patterns in the cohort children who had been admitted to hospital with severe malaria were compared to the profiles in children of the same age, residential location and time of sampling. Although the background cumulative parasite rates were relatively low, detection of antibodies to schizont extract suggested an overall high exposure to *P. falciparum* parasites in both cases and controls. This high seroprevalence confirms that a majority of cases had been exposed to *P. falciparum* prior to the severe disease episode, thus arguing against the notion that severe malaria occurs predominantly after first encounters with the parasite.

The three-monthly assessments provide only snapshots of the infection dynamics [22] and data were not available on the incidence of mild malaria and the frequency of antimalarial treatment. The number of infections in these children is thus likely to be underestimated. One interpretation of the higher prior parasite positivity among the cases is that this simply reflects higher exposure, due to local heterogeneity in transmission, i.e. “hot spots” [23].

However, the finding of similar and high rates of preexisting antimalarial antibodies in the closely matched cases and controls indicates that similar proportions of children have been exposed to *P. falciparum* at least once. In the youngest children, antibodies might have been partly maternally derived and a more in depth analysis of the dynamics of anti-malarial antibodies in these children is ongoing.

An alternative explanation for the more frequent prior detection of parasites in children developing severe malaria is that it reflects less good control of parasite densities in those individuals following exposure to malaria. It should be noted that most prior infections were asymptomatic at the cohort visits suggesting a state of partial immunity, at least to the infecting parasite. Children thus succumb to severe malaria despite having previously been able to harbor parasites without fever. We therefore tested whether subsequent episodes of severe disease represented exposure to new infections.

Genotyping of the *msp2* gene provides more sensitive and detailed data on different *P. falciparum* populations than just assessing parasite prevalence by microscopy or PCR. The genotyping confirmed that the infections at the time of the severe malaria admissions were all different from the parasites detected in previous three-monthly visits. The finding of multiclonal *P. falciparum* infections in the cohort visits might be a result of several genotypes transmitted in single mosquito inoculations but can also reflect accumulation of repeated infections. However, most PCR positive samples in these children were composed of single clones. Also in high transmission areas where infections are mainly multiclonal, children younger than 1 year are more often infected with fewer clones [24], [25] and duration of infections and number of concurrent clones increase with age [25], [26].

The risk of severe malaria was highest in children who had a multiclonal infection detected in at least one sample since birth. In previous studies of uncomplicated malaria from this and other areas of low or moderate transmission intensity, multiclonal infections in asymptomatic individuals at base line have predicted an increased risk of subsequent malaria [27]-[29]. In high transmission settings and in older children detection of multiple *P. falciparum* clones has, on the contrary, been associated with a reduced risk of uncomplicated malaria [29], [30]. The contradictory findings could be explained by the idea that in young children with limited acquired immunity, multiple clones reflect either higher degrees of exposure to malaria or less good immune control. As argued above, data on antibody prevalence suggest the latter. In a recent study, the cumulative number of clones in repeated samples, defined as “molecular force of infection”, was found to be a major factor determining the risk of clinical malaria in children up to three years of age and was suggested as measure of individual exposure [31]. How and when the transition from susceptibility to protection against clinical malaria occurs in an individual with regards to exposure to different parasite variants remains to be established.

The design of this study was unique in the sense that the three-monthly sampling within this large birth cohort allowed for genotyping of the infections encountered before the episodes of severe malaria. Although the study was running over several years, only a limited number of children had developed severe malaria within the cohort. Since Kilifi District Hospital is the only referral hospital in the district and the systematic recording of all pediatric hospital admissions is well established, we believe that the linkage was successful in capturing the majority of admissions of severe malaria within the cohort. Only including children with the major syndromes of severe malaria restricted the number of cases, however a high specificity was achieved [4]. The relatively low number of cases identified within the cohort reflects the decline of malaria transmission in the study area [15]. Matching for location, age and season (+/−3 months) was thus highly important to control for regional and temporal differences in transmission in Kilifi District during the study period.

Immunity to severe non-cerebral malaria has been suggested to be acquired after only one or two infections [3]. Here, none of the cases were parasite positive in more than two three-monthly samples before admission. Nonetheless, up to six different clones were detected over time before admission in children with severe non-cerebral malaria. In contrast, the children who developed severe malaria with impaired consciousness never had more than one clone prior to admission, thus suggesting different force of infection. Further analysis with regards to the different severe malaria syndromes was limited by the number of cases for subgroup analysis. Nonetheless in addition to having no detected prior multi-clonal infections, children with impaired consciousness also were less likely than children with other forms of severe disease to have evidence of exposure as judged by antimalarial antibodies. Children admitted with impaired consciousness might be those who either developed severe complications during their first infection or those that did not acquire appropriate immune responses during earlier infections.

Knowledge of how and when children develop protective immunity to severe malaria is important for the development of a vaccine and important also for design of other control measures. Although findings need to be confirmed, the results suggest that infection patterns differ between children who develop severe malaria when compared with children who do not, and also may differ in children who develop different syndromes of severe malaria. Although epidemiological evidence shows that immunity develops faster to severe than to mild disease, our results argue against the notion that children who succumb to severe malaria are previously naïve to the infection.

## Supporting Information

Figure S1 **Distribution and mean age at admissions with severe malaria within the cohort during the study period 2002–2010.** Severe malaria admissions were included up to 2010, i.e. 2 years after inclusions ended.
(TIF)

Figure S2 **Distribution of alleles of A) FC27 types and B) IC types in admissions and three-monthly visits.** The y-axes determine the number of alleles of the same fragment length at base pair precision.
(TIF)

File S1 **Additional information of** ***msp2*** **genotyping profiles and allele frequencies in visits and admissions.**
(DOCX)
