# Supplementary figures and images for: Plasmodium falciparum Infection Patterns Since Birth and Risk of Severe Malaria: A Nested Case-Control Study in Children on the Coast of Kenya
Source: PLoS One. 2013 Feb 13;8(2):e56032. doi: 10.1371/journal.pone.0056032 (PMC3572150; doi:10.1371/journal.pone.0056032)

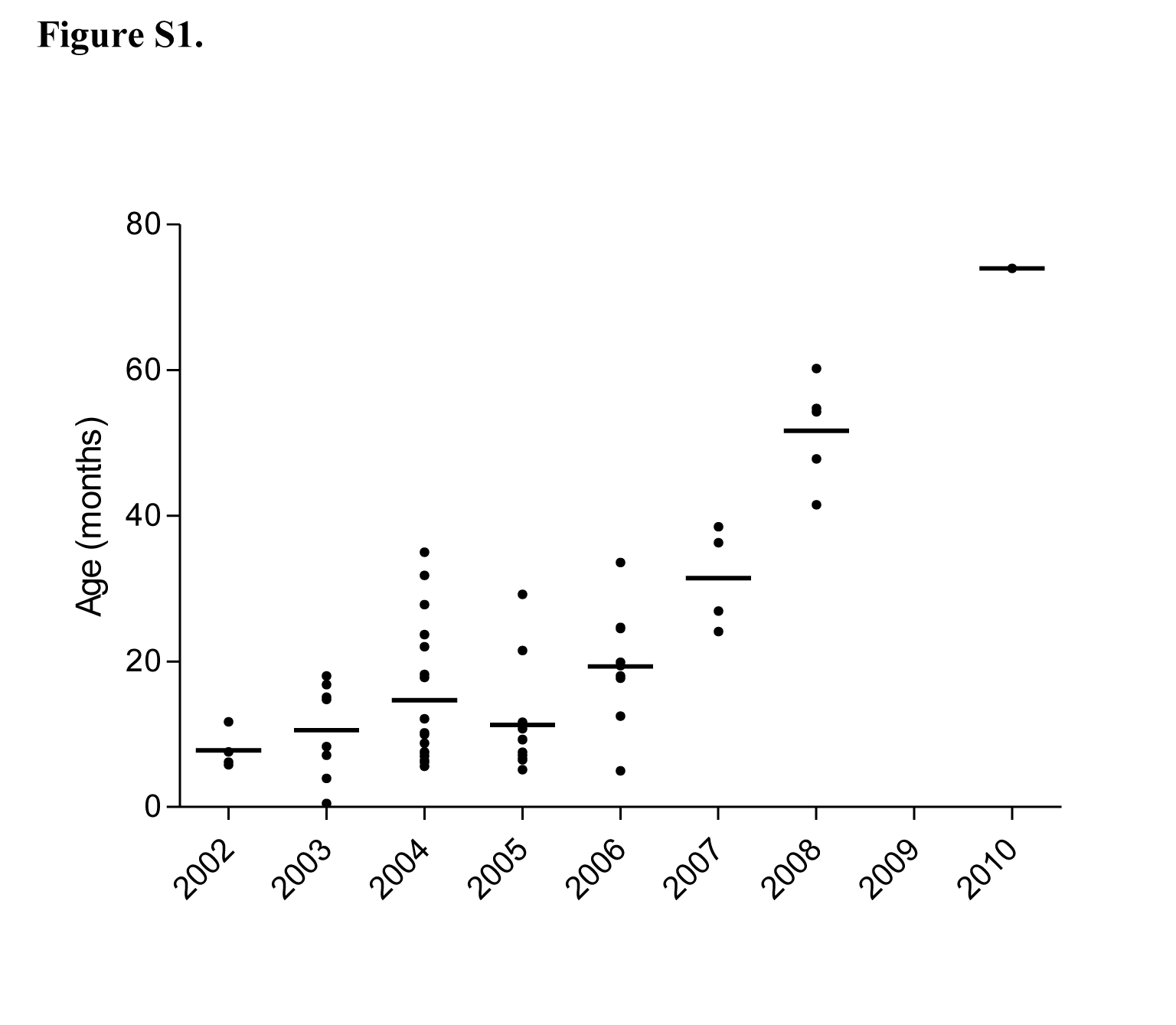

Supplement: Figure S1 — Distribution and mean age at admissions with severe malaria within the cohort during the study period 2002–2010. Severe malaria admissions were included up to 2010, i.e. 2 years after inclusions ended. (TIF) [file pone.0056032.s001.tif]

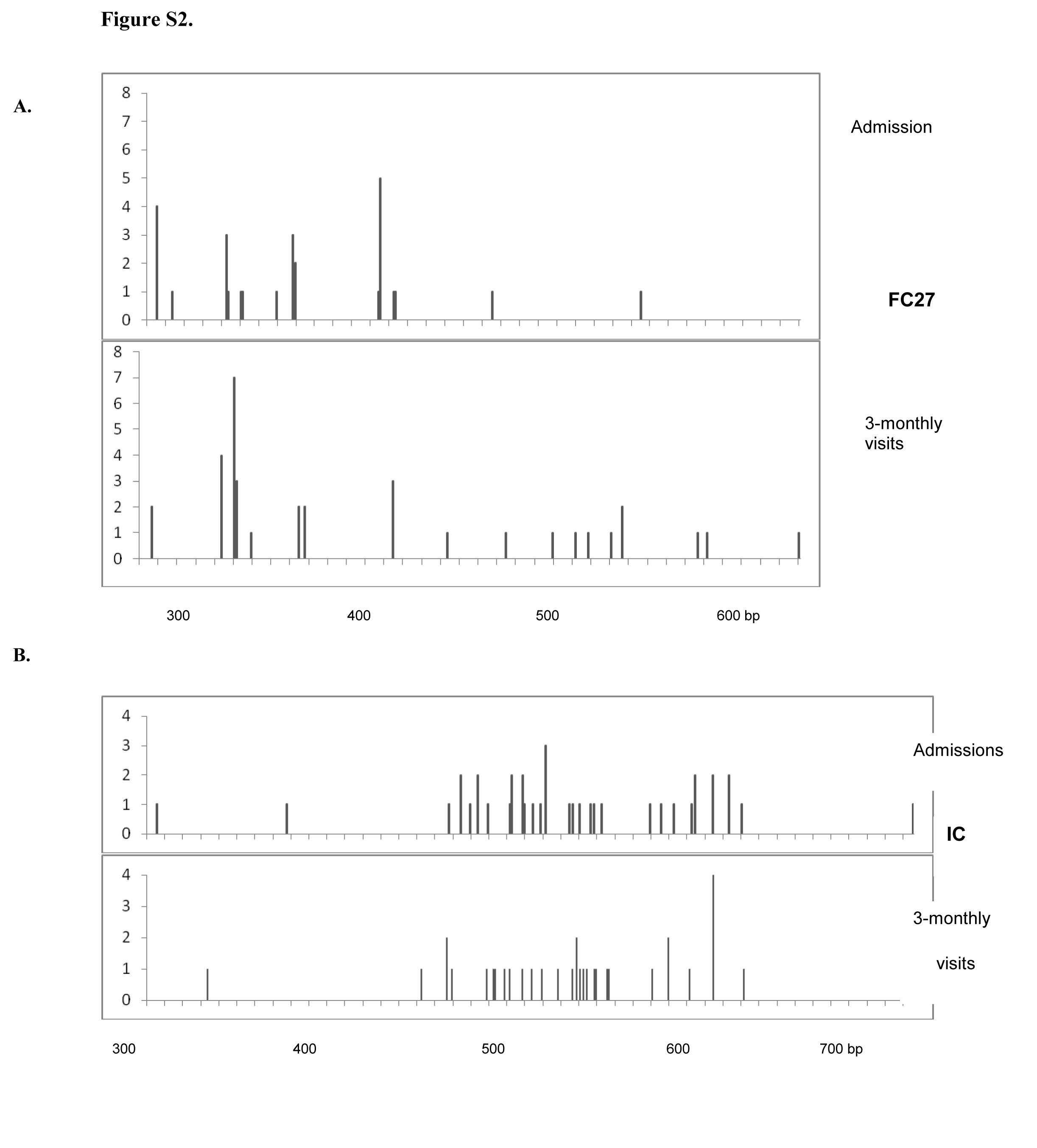

Supplement: Figure S2 — Distribution of alleles of A) FC27 types and B) IC types in admissions and three-monthly visits. The y-axes determine the number of alleles of the same fragment length at base pair precision. (TIF) [file pone.0056032.s002.tif]
