# Supplementary material for: Plasmodium falciparum Infection Patterns Since Birth and Risk of Severe Malaria: A Nested Case-Control Study in Children on the Coast of Kenya
Source: PLoS One. 2013 Feb 13;8(2):e56032. doi: 10.1371/journal.pone.0056032 (PMC3572150; doi:10.1371/journal.pone.0056032)
Supplement: File S1 — Additional information of msp2 genotyping profiles and allele frequencies in visits and admissions. (DOCX) [file pone.0056032.s003.docx]

**Supporting information**

**Genotyping of *P. falciparum* *msp2* in three-monthly visits and admissions**

Detection of *P. falciparum* parasites in the 667 three-monthly visits in cases and controls was higher by PCR (6.9%) than by microscopy (4.5%). The sensitivity of detection of the PCR assay was 1-10 parasites /µl whole blood. Five samples were positive by microscopy and negative by PCR. Parasite densities in asymptomatic visits were 300-355 000 p/µl (mean 155150 p/µl). Fever (axillary temperature >37.5 °C) was recorded at 36 (5%) of the visits of which 12 (33%) were malaria positive by microscopy (1540-410000 p/µl, mean 189308 p/µl) (Table 1).

A cut off of 150 relative fluorescent units was applied when analyzing PCR products to avoid unspecific background artifacts, as optimized in the laboratories at KEMRI in Kilifi. The IC (3D7) type was the most prevalent of the *msp2* alleles in three-monthly visits with 42% of infections with only IC alleles, 14% only FC27-alleles and 44% composed of both types. In total 50 IC alleles of same base pair length and 37 when binned into 3 bp bins were detected (size range 462-720 bp); and 33 FC alleles (size range 291-635 bp) and 27 when into 3bp. In 5 of the total 83 alleles the same base pair length, and 11 of 64 alleles after 3 bp binning, were detected both in visit and admission samples (Figure S2). The *msp2* genotypes were named according to their allelic type (FC27 or IC) followed by the length of the amplified fragment, e.g. FC410 determined 410 bp long FC27 fragments. FC335 was found in 10 samples from cohort visits and 1 severe malaria episode, from children living in 5 different locations.

In three children (all controls and asymptomatic) parasites with the same *msp2* allelic families and exact same fragment lengths were detected more than once with several months interval; two children had single alleles detected with 3 and 6 months interval. A third child had in total 11 different alleles of which 4 were detected in two samples 9 months apart; in addition *P. malariae* was detected in two three-monthly consecutive samples (Table S1).

A blood sample collected at the time of the severe malaria admission was available from 40 of the 61 cases. Parasite densities ranged from 160-1540800 p/µl (mean 168194 p/µl). Genotyping revealed that single *msp-2* alleles were detected in 21 (52%) of the acute severe infections, 2 alleles in 15 (38%) and 3 alleles in 4 children (10%). Of the two allelic types of *msp2*, the IC type was detected alone in 35% of samples, the FC 27 type in 17% samples; and alleles of both types were detected in 48% of the samples. Most genotypes were present in only one severe malaria infection, however, FC 363 and FC 410 appeared in 5 patients each (with different syndromes), and never in asymptomatic individuals at cohort visits. These samples were all from different locations and time points, i.e. showed no signs of clustering.

**Table S1. Genotyping and microscopy profiles of three-monthly samples from birth in child 4784^a^ (control)**

| **KBC4784** | | **Age (months)** | | | | | | |
| --- | --- | --- | --- | --- | --- | --- | --- | --- |
|  |  | **3** | **6** | **9** | **12** | **15** | **18** | **21** |
| **Microscopy** | ***P. falciparum* (p/ul)** | 0 | 0 | 0 | 0 | 140000 | 600 | 50000 |
|  | **Other species** |  |  |  |  |  | ***P. malariae*** | ***P. malariae*** |
| ***P. falciparum***  ***msp2***  **genotyping** | **FC27 alleles^b^ (bp)** | 0 | **291**  **328**  **364** | 0 | 0 | **291**  **328**  **364** | 344 | nd |
|  | **IC alleles^b^**  **(bp)** | 0 | 478  497  **503** | 372 | 0 | **503** | 469  516  597 | nd |
|  | **Total** | 0 | 6 | 1 | 0 | 4 | 4 | nd |
| **Clinical status** | **Fever** | 0 | 0 | 0 | 0 | 0 | 0 | 0 |

1. This child was not admitted to hospital at any time during the study period, nor had fever at any of the three-monthly visits
2. Alleles of same fragment lengths in different samples in bold.
